# Hydroponic Chinese flowering cabbage detection and localization algorithm based on improved YOLOv5s

**DOI:** 10.1371/journal.pone.0315465

**Published:** 2024-12-16

**Authors:** Zhongjian Xie, Yaya Zhang, Weilin Wu, Yao Xiao, Xinwei Chen, Weiqi Chen, ZhuXuan Wan, Chunhua Lin

**Affiliations:** 1 School of Physics and Electronic Information, Guangxi Minzu University, Nanning, China; 2 Guangxi Key Laboratory of Machine Vision and Intelligent Control, Wuzhou University, Wuzhou, China; Government College University Faisalabad, PAKISTAN

## Abstract

To achieve automated harvesting of hydroponic Chinese flowering cabbage, the detection and localization of the cabbage are crucial. This study proposes a two stages detection and localization algorithm for hydroponic Chinese flowering cabbage, which includes macro-detection and micro-localization. The macro-detection algorithm is named P-YOLOv5s-GRNF. Its improvement strategies include adopting pruning techniques, the GSConv, receptive field attention convolution (RFAConv), normalization-based attention module (NAM), and the Focal-EIOU Loss module. The micro-localization algorithm is named YOLOv5s-SBC. Its improvement strategies include adding a 160×160 detection layer, removing a 20×20 detection layer, introducing a weighted bidirectional feature pyramid network (BiFPN) structure, and utilizing the coordinate attention (CA) mechanism. The experimental results showed that P-YOLOv5s-GRNF increased the mAP(mean average precision) by 0.8%, 4.3%, 3.2%, 0.7%, 19.3%, 9.8%, 3.1% compared to mainstream object detection algorithms YOLOv5s, YOLOv6s, YOLOv7-tiny, YOLOv8s, YOLOv5s-Shufflenetv2, YOLOv5s-Mobilenetv3, YOLOv5s-Ghost, respectively. Compared to the original model, P-YOLOv5s-GRNF decreased parameters by 18%, decreased model size to 11.9MB, decreased FLOPs to 14.5G, and increased FPS by 4.3. YOLOv5s-SBC also increased mAP by 4.0% compared to the original YOLOv5s, with parameters decreased by 65%, model size decreased by 60%, and FLOPs decreased to 15.3G. Combined with a depth camera, the improved models construct a positioning system that can provide technical support for the automated and intelligent harvesting of Chinese flowering cabbage.

## 1 Introduction

Chinese flowering cabbage [1], one of the commonly consumed vegetables in China, is highly favored by consumers due to its unique flavor, rich nutrition, and various health benefits. Its cultivation area gradually expanding, with plantings observed in areas such as Guangdong Province, Guangxi Province, Ningxia Province, and Yunnan Province. In recent years, with the increasing scarcity of arable land and the growing demand for high-quality vegetables, plant factories utilizing hydroponic methods have been on the rise. The cultivation of hydroponic Chinese flowering cabbage is not affected by seasons or locations, freeing it from resource and environmental constraints. This method can effectively alleviate the problem of arable land scarcity, achieve industrialized agricultural production, and significantly increase productivity. Therefore, the development of the Chinese flowering cabbage industry holds promising prospects for the future.

As a future direction in planting technology, the production of hydroponic Chinese flowering cabbage involves several vital processes: sowing, seedling cultivation, transplanting, and harvesting. Among these, harvesting stands out as particularly labor-intensive. Currently, Chinese flowering cabbage is predominantly harvested manually, which incurs substantial labor costs, thus hindering the industry’s development. The advancements in machine vision and deep learning provide substantial support for agricultural automation and smart healthcare, among other fields. There has been extensive research on fruit and vegetable harvesting equipment [2–5] globally; however, the predominant focus has been on fruit harvesting, with scant attention devoted to harvesting leafy stem vegetables.

In the research on harvesting hydroponic leafy vegetables, Ling [6] utilizes a pneumatic cylinder to grip and cut the stems of the hydroponic plants. A push rod mounted on the cutting device facilitates the transfer of harvested leafy vegetables onto a conveyor belt, thereby completing the harvesting process. While this method enhances harvesting efficiency, it also poses challenges in cleaning the remaining roots within the pipeline.

There is extensive research on visual detection [7–9] in fruit harvesting equipment. Wang et al. [10] proposed a lightweight real-time detection method for apples. They adopted YOLOv4 as the foundational framework, MobileNetv3 as the feature extraction network, integrated depth-wise separable convolution, and introduced a coordinate attention mechanism. Their approach achieved an average precision (AP) of 92.23% with a detection speed of 15.11 frames per second (f/s) on embedded platforms. Zhang et al. [11] focused on tomato fruit detection and improved YOLOv5s by incorporating a convolutional attention module and utilizing CIoU Loss as the loss function. They attained an impressive mAP of 99.53%. Liu [12] improved the detection precision of rapeseed seedlings by employing a customized YOLOv3 model. Their approach involved substituting the residual network with a densely connected network and refining the loss function, resulting in an AP of 93.44%. Huang et al. [13] introduced a lightweight algorithm for recognizing tea buds, leveraging improvements to the YOLOv4 model. They replaced the original backbone network with GhostNet, integrated Ghost convolution within the neck network, and utilized transfer learning techniques. Their method achieved mAP of 72.93%. Yao et al. [14] developed a YOLOv5-based kiwifruit defects detection model. The model was improved by adding a small target detection layer, introducing SELayer, training the model using transfer learning, and applying the Cosine Annealing algorithm to enhance performance. The improved model achieved mAP of 94.7%. Huang et al. [15] proposed a lightweight algorithm for strawberry detection. They replaced the original YOLOv5s backbone with the MobileNetV3 network, introduced the Alpha-IoU loss, and used the K-Means++ algorithm to cluster the original YOLO anchors. They also added detection heads to improve the detection accuracy of small targets. The improved network model achieved a detection frame rate of 44 f/s, which is a 15.7% improvement over the original model, with a mAP of 99.4%.

Qi et al. [16] proposed an improved SE-YOLOv5 network model for tomato virus diseases by adding a squeeze-and-excitation module to the YOLOv5 model, drawing inspiration from the human visual attention mechanism. The experimental results showed a mAP of 94.10%. Gu et al. [17] proposed an improved YOLOv5 model for seed potato bud eye detection. The model incorporates the C3 Faster module, the gather-and-distribute module, and improved loss function. They also optimized hyperparameters using a genetic algorithm, followed by pruning and distillation. The improved model has 57.1% of the parameters of the original model, with a mAP of 90.5%.

In terms of object localization [18–20], Li et al. [21] focused on detecting and accurately locating mulberry tree branches. They improved the YOLOv5 model by incorporating the Convolutional Block Attention Module (CBAM), adding a small object layer, and refining the loss function. By combining this with a depth camera, they performed coordinate transformations to obtain three-dimensional coordinates. Guo et al. [22] proposed an object detection and localization algorithm to address the challenge of low precision in safflower flower crown detection. They employed an improved YOLOv5 model and a mobile camera-based localization method, achieving a successful picking rate of 90.20%. Yang et al. [23] improved the YOLOv3 model for Citrus to enhance the model’s ability to extract features of branches and leaves. They used a depth camera to obtain 3D information on the harvesting target. The detection and localization system achieved a detection rate of 91.9% for harvestable fruits, with a harvesting success rate of 80.51%. Duan et al. [24] utilized an improved YOLOv5 algorithm model, combined with a depth camera, to localize the the fruit axis at the bottom of banana, They incorporated the Coordinate Attention module and replaced the CIoU loss with the EIoU loss. In the localization experiments, the mean error of the improved model was 0.063m. Wang et al. [25] proposed an improved YOLOv5s model for camellia oleifera fruit detection. The model was enhanced by adding a small target detection layer, embedding the Faster Block module in the C3 module, and introducing the Biformer attention mechanism. The improved model was deployed on the Jetson Xavier NX and combined with a depth camera for camellia oleifera fruit detection and localization experiments. The recall rate of the experiment was 91.7%.

The aforementioned research suggests that combining the YOLO algorithm with a depth camera [26, 27] is effective for detecting and localizing objects in fruit and vegetable harvesting. However, challenges remain in optimizing model efficiency and improving localization, especially in the context of detecting hydroponic Chinese flowering cabbage. Currently, there are no automated harvesting devices specifically developed for hydroponic Chinese flowering cabbage, and there are no existing detection models designed for this purpose in China. Further research in these areas is needed.

This study focuses on the detection and localization algorithm for hydroponic Chinese flowering cabbage. The algorithm follows a two stages process: macro-detection and micro-localization. Initially, an improved P-YOLOv5s-GRNF model is employed to detect the entire Chinese flowering cabbage. Subsequently, an improved YOLOv5s-SBC model is utilized to identify the root area. Finally, the improved root area detection model is integrated with a depth camera system to achieve detection and localization of hydroponic Chinese flowering cabbage.

The main contributions of this study are summarized as follows:

1. A model structure suitable for Hydroponic Chinese flowering cabbage detection and localization is restructured composing by macro-detection and micro-localization two stages algorithm.
2. Improvements of macro-detection algorithm are made by adopting pruning techniques, the GSConv, receptive field attention convolution (RFAConv), normalization-based attention module (NAM), and the Focal-EIOU Loss module.
3. Optimizations of micro-localization algorithm are made by adding a small object detection layer, removing a large object detection layer, introducing a weighted bidirectional feature pyramid network (BiFPN) structure, and utilizing the coordinate attention (CA) mechanism.
4. Localization experiments combined with a depth camera were demonstrated, proving the model proposed in this study is high detection performance.

## 2 Materials and methods

### 2.1 Image acquisition and preprocessing

The Images of hydroponic Chinese flowering cabbage were collected in the laboratory at Guangxi Minzu University. A depth camera, Intel D435i, was utilized as the image acquisition device. Photographs were captured from diverse angles and distances to encompass various perspectives of Chinese flowering cabbage, including scenarios where individual and multiple plant leaves overlapped. 618 images were gathered for the dataset of entire hydroponic Chinese flowering cabbage. Additionally, 508 images were specifically collected for the dataset focusing on the root area. All images are in JPG format and have a resolution of 1920×1080 pixels.

To improve the generalization ability of the detection and localization algorithm for hydroponic Chinese flowering cabbage, image enhancement operations such as adjusting brightness and adding noise were applied to the images. The final dataset for the entire hydroponic Chinese flowering cabbage comprises 1236 images. These images were manually annotated using the LabelImg tool in PASCAL VOC format and saved as XML files. The images were randomly divided into training (989 images), validation (123 images), and test sets (124 images). Additionally, images of the root area underwent similar preprocessing steps. This dataset consists of 1,016 images, further divided into training (813 images), validation (101 images), and test sets (102 images).

### 2.2 Improvement of the macro-detection algorithm

#### 2.2.1 Baseline model selection

There are two types of object detection algorithms based on deep learning: one-stage and two-stage. One-stage object detection algorithms generally exhibit faster detection speeds compared to their two-stage counterparts. YOLO, as a prominent one-stage object detection algorithm, balances high detection precision with rapid computation speeds.

Currently, YOLOv5 is available in five versions: YOLOv5n, YOLOv5s, YOLOv5m, YOLOv5l, and YOLOv5x. While these versions share the same network architecture, they vary in terms of width and depth. The model size, number of parameters, and floating-point operations (FLOPs) increase sequentially from YOLOv5n to YOLOv5x. Table 1 displays their mAP (mean average precision) on the entire hydroponic Chinese flowering cabbage dataset, which are 94.1%, 95.9%, 97.0%, 97.0%, and 97.2%, respectively. Taking into account parameters, detection precision, and other considerations, this study selects YOLOv5s for further enhancement.

**Table 1:** Comparative experiment of different YOLOv5 versions.

Model	mAP (%)	Size (MB)	Parameters	FLOPs (G)
YOLOv5n	94.1	3.9	1760518	4.1
YOLOv5s	95.9	14.4	7012822	15.8
YOLOv5m	97.0	42.2	20852934	47.9
YOLOv5l	97.0	92.8	46108278	107.6
YOLOv5x	97.2	173.1	86173414	203.8

**Fig 1:**
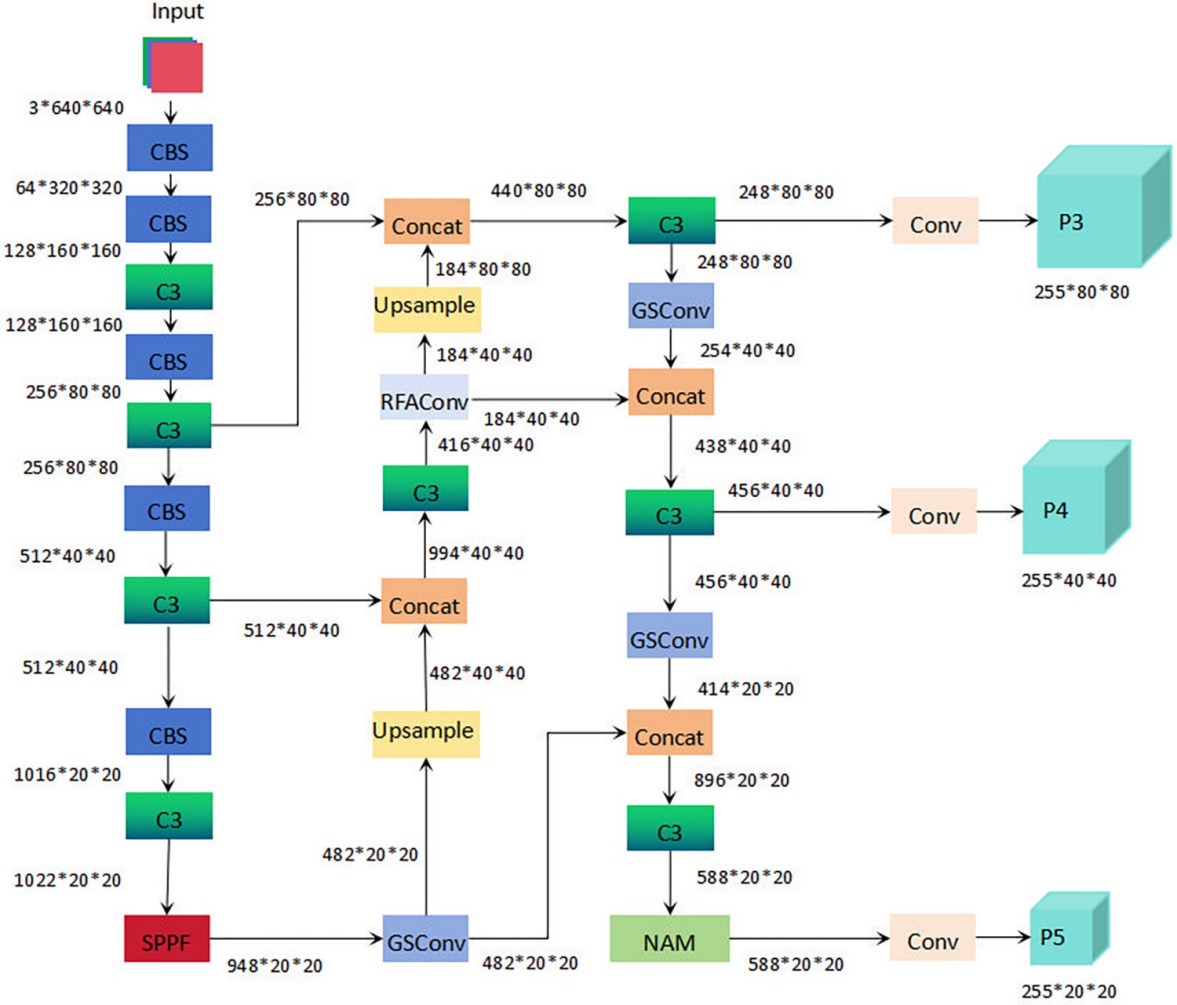
Structure of P-YOLOv5s-GRNF. (1) 64*320*320: 64 represents the number of channels, and 320*320 represents the image size.

Enhancements were made to the YOLOv5s model, including pruning [28] to decrease the parameter count while preserving the original precision, the integration of the GSConv [29] module for more efficient parameter management, the addition of the RFAConv [30] module to extract crucial image details and enhance detection precision, and the incorporation of the NAM [31] mechanism. Additionally, the model employed Focal-EIOU Loss [32] instead of CIOU Loss [33] to further improve detection precision. This improved model is referred to as P-YOLOv5s-GRNF, with its architectural details illustrated in Fig 1.

#### 2.2.2 Pruning of the YOLOv5s Model

**Fig 2:**
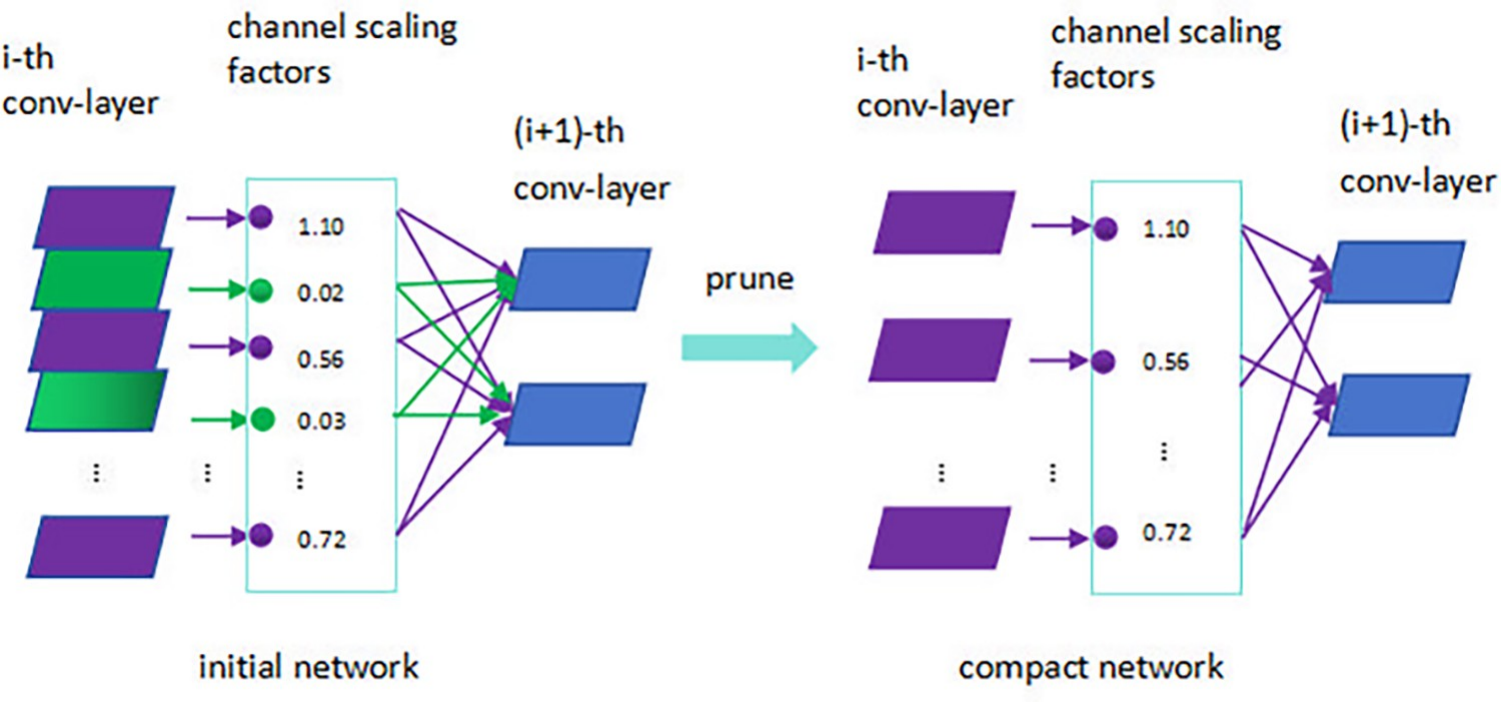
Pruning process.

The pruning technique reduces the size and FLOPs of a model by eliminating redundant parameters while maintaining detection precision. The process involves sparsifying the training, setting an appropriate sparsity rate λ, introducing a scaling factor γ for each channel, and regularizing the sparsity of these scaling factors. As the scaling factor γ for each channel approaches zero, these channels are identified as less important and are thus pruned. It is essential to choose an appropriate pruning rate; a rate that is too low may result in only minimal reductions in model size, while a rate that is too high can severely impair model performance. After pruning, a slight decrease in model precision may occur, but this can be restored through fine-tuning. The pruning process is illustrated in Fig 2.

#### 2.2.3 GSConv module

To further reduce the parameters and enhance the detection speed of the entire hydroponic Chinese flowering cabbage detection model, a strategy involving the substitution of regular convolutions with lightweight alternatives can be implemented. However, many lightweight models [34] rely solely on depth-wise separable convolutions, which often result in notable decreases in detection precision. This research introduces a novel convolutional module known as GSConv. It utilizes channel random mixing to combine regular convolutions with depth-wise separable convolutions, aiming to lower model complexity while maintaining detection precision. This research proposes integrating GSConv into the Neck network of YOLOv5s, replacing the 10th, 18th, and 21st regular convolutions.

#### 2.2.4 RFAConv module

**Fig 3:**
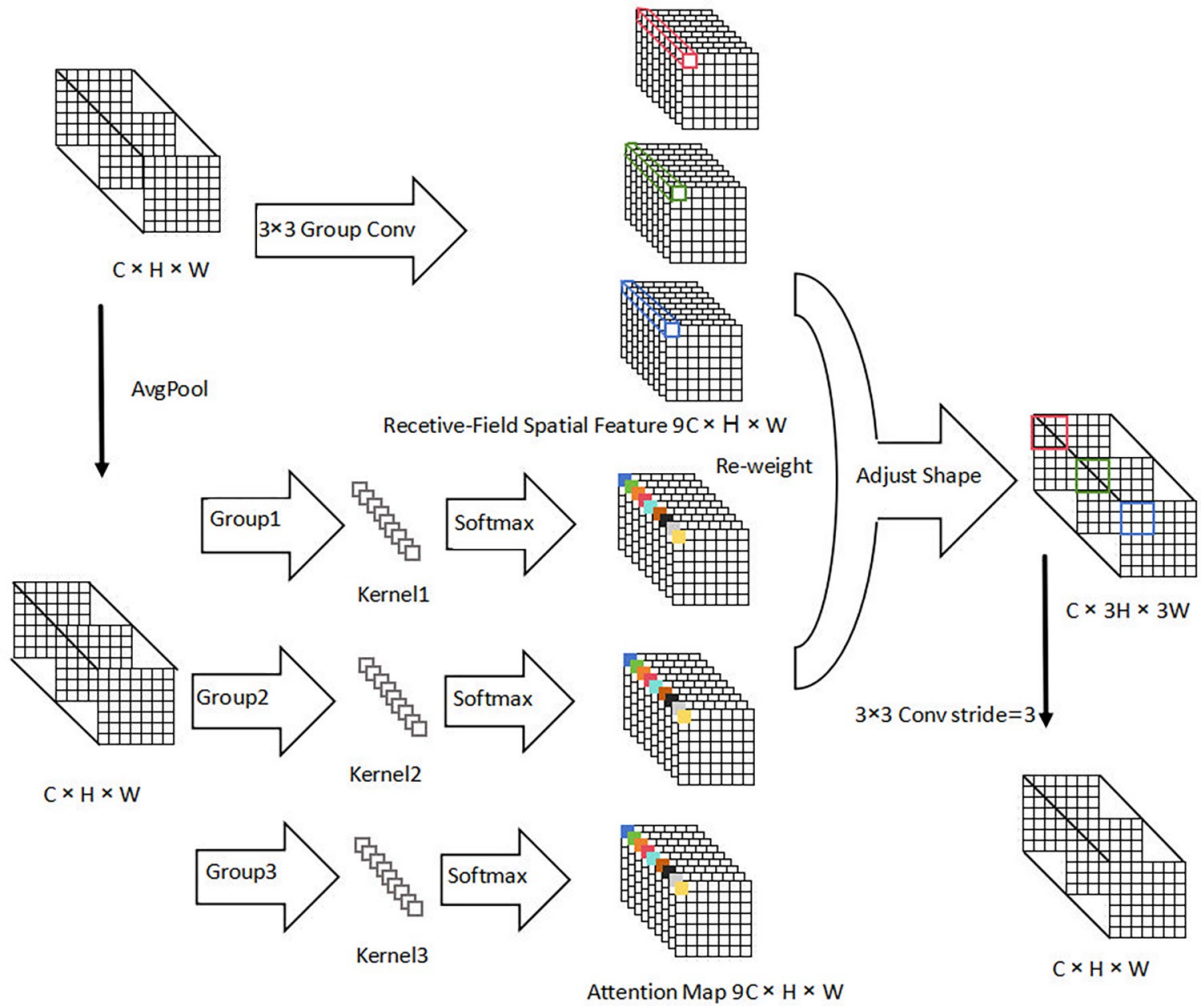
Principle and structure of RFAConv.

In YOLOv5s, standard convolutions extract feature information from different image positions using shared parameters, overlooking variations in feature importance across positions. This limitation adversely affects model performance, especially in scenarios with densely distributed objects or low-light conditions. This study introduces RFAConv to address parameter sharing in convolutional kernels. RFAConv assigns distinct parameters to kernels based on varying image features across areas, thereby focusing on spatial aspects of the receptive field and processing information differently across areas. By applying effective attention weights to large kernels, RFAConv efficiently captures crucial image details. While this may marginally increase FLOPs and parameters, it enhances the average precision of the hydroponic Chinese flowering cabbage detection model overall. The principle and structure of RFAConv are illustrated in Fig 3, where AvgPool extracts global information from all receptive field features, and softmax highlights the significance of each feature in enhancing network performance.

#### 2.2.5 NAM module

In order to improve the precision of model detection by reducing the influence of less significant weights, this study introduces a Normalization-based Attention Module (NAM) into the Neck network as a lightweight attention mechanism. Drawing inspiration from CBAM [35], the Channel and Spatial Attention Sub-modules of the NAM are redesigned. The integration of Batch Normalization’s (BN) scaling factor serves to augment and fine-tune the attention mechanism.

#### 2.2.6 Improvement of loss function

The default loss function in YOLOv5s is CIOU Loss. This function represents an evolution from IOU Loss, GIOU Loss [36], and DIOU Loss [37], reflecting ongoing improvements in loss function optimization. CIOU Loss enhances previous methods by incorporating considerations such as the overlap area between the detection box and the target box, the distance between the center points of the bounding boxes, and the aspect ratio. However, CIOU Loss primarily addresses differences in aspect ratios and does not account for the balance between high-quality and low-quality samples. In this study, detecting entire hydroponic Chinese flowering cabbage faces challenges such as leaf occlusion and dense detection objects, making CIOU Loss insufficient for optimal detection results. Therefore, this study adopts Focal-EIOU Loss. EIOU Loss reduces the discrepancy in aspect ratios between predicted and ground-truth boxes, accelerating convergence and enhancing regression precision, as defined in Eq 1. Furthermore, Focal Loss is employed to improve the balance between high-quality and low-quality samples, as specified in Eq 2.


$$\begin{array}{l} L_{EIOU}=L_{IOU}+L_{dis}+L_{asp}\\ \;=1-IOU+\frac{\rho^{2}(b,b_{gt})}{w_{c}^{2}+h_{c}^{2}}+\frac{\rho^{2}(h,h_{gt})}{h_{c}^{2}}+\frac{\rho^{2}(w,w_{gt})}{w_{c}^{2}} \end{array}$$ (1)

$$L_{Focal-EIOU}={IOU}^{\gamma }L_{EIOU}$$ (2)

In the equation, *L*_*IOU*_ represents the IOU loss, *L*_*dis*_ represents the center distance loss, and *L*_*asp*_ represents the aspect ratio loss, *b* represents the center point of the predicted box, *b*_*gt*_ represents the center point of the ground-truth box, *ρ* represents the Euclidean distance between points *b* and *b*_*gt*_, *h* and *w* respectively represent the height and width of the predicted box, *h*_*gt*_ and *w*_*gt*_ respectively represent the height and width of the ground-truth box, *h*_*c*_ and *w*_*c*_ respectively represent the height and width of the minimum enclosing rectangle that bounds both the predicted box and the ground-truth box, *γ* is a parameter controlling the degree of suppression of outliers.

### 2.3 Improvement of micro-localization algorithm

#### 2.3.1 Algorithm optimization

**Fig 4:**
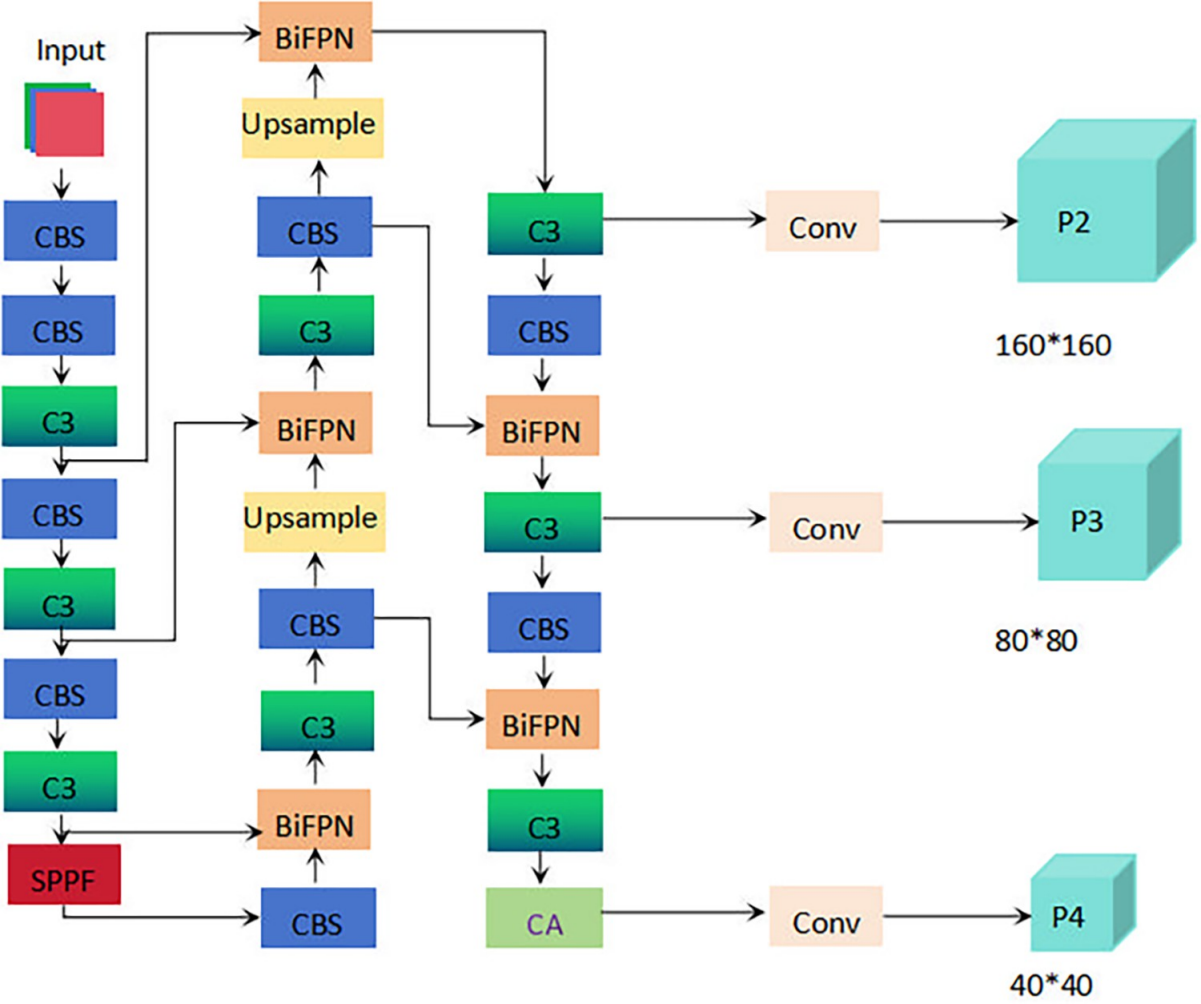
Structure of YOLOv5s-SBC.

The baseline network selected for the root area of hydroponic Chinese flowering cabbage was also YOLOv5s. Enhancements were applied to the YOLOv5s model, including adding a small object detection layer, removing a large object detection layer, and reducing parameters, which resulted in improved original precision of the model. Furthermore, the model incorporated a weighted BiFPN [38] and introduced the CA [39] mechanism to enhance detection precision further. The improved model is named YOLOv5s-SBC, and its structure is illustrated in Fig 4.

#### 2.3.2 Lightweight improvement of the network structure

This study delineates and analyzes the distribution of width-to-height ratios within the root area of hydroponic Chinese flowering cabbage dataset labels, suggesting that these are categorized as small objects for detection. YOLOv5s employs an 80×80 feature map size to detect small objects; however, this may not effectively capture even smaller objects. To enhance detection of such smaller objects, this study increases the feature map size to 160×160, thereby improving the model’s precision. Conversely, the feature map size of 20×20, which is utilized for detecting larger objects, does not enhance the model’s precision when removed. Instead, its removal increases the model’s parameters and FLOPs. Therefore, the 20×20 feature layer, along with layers 7 and 8 of the backbone network, are removed.

#### 2.3.3 BiFPN module

To address the challenge of low precision in detecting the root area of hydroponic Chinese flowering cabbage, the path aggregation network (PaNet) layer in YOLOv5s is substituted with the BiFPN layer. Compared to PaNet, BiFPN functions serve as a weighted bidirectional feature pyramid network. It enhances feature fusion across various levels by employing top-down, bottom-up, cross-scale, and skip connections. This approach improves the representation of shallow feature information, thereby increasing the precision of the root area of hydroponic Chinese flowering cabbage.

#### 2.3.4 CA module

To further improve the detection precision of the root area of hydroponic Chinese flowering cabbage, this study incorporates the Coordinate Attention (CA) mechanism to address the limitations of conventional attention mechanisms like SE [40] in capturing coordinate information. The CA mechanism integrates positional information into channel attention through two main steps: embedding coordinate information and generating coordinate attention. To overcome the challenge of retaining positional information with single global average pooling, the process is divided into horizontal and vertical encoding. This approach captures long-range dependencies in one spatial direction while maintaining precise positional information in the other, thereby improving the model’s precision in locating the root area.

## 3 Experimental design and results analysis

### 3.1 Experimental environment and parameter settings

The experiment was conducted on a laptop running Windows 11, equipped with a 12th Gen Intel® Core™ i5-12500H CPU at a base frequency of 3.10 GHz. Computational acceleration was provided by an NVIDIA GeForce RTX 3060 GPU with 6GB of memory. The development environment used PyTorch deep learning framework, version 1.12.1, and Python 3.8, with CUDA version 12.0. For model training, the initial learning rate was 0.01 with a momentum of 0.937, and the total number of epochs was 300. The batch size for the improved YOLOv5s model detecting entire hydroponic Chinese flowering cabbage was set to 4, while for detecting the root area of the same, it was set to 16.

### 3.2 Model evaluation metrics

A comprehensive set of widely recognized evaluation metrics is utilized to assess the performance of the P-YOLOv5s-GRNF and YOLOv5s-SBC algorithms in the domain of object detection and localization. The evaluation metrics include mean average precision (mAP), model size, number of parameters, frames per second (FPS), and computational complexity measured in giga floating-point operations per second (GFLOPs). The equations are as follows.


$$P=\frac{TP}{TP+FP}$$ (3)

$$R=\frac{TP}{TP+FN}$$ (4)

$$AP=\int_{0}^{1}P(R)dR$$ (5)

$$mAP=\frac{1}{n}\sum_{k=1}^{n}AP_{k}$$ (6)

Where, P represents precision and R represents recall, TP, FP and FN respectively represent true positive, false positive and false negative, AP represents the area under the precision-recall curve, n represents the number of classes.

### 3.3 YOLOv5s model pruning

During sparsity training, it is crucial to set an appropriate sparsity rate λ. If λ is too low, the scaling factor γ will not approach zero, resulting in insufficient sparsity and only a limited proportion of parameters being pruned. Conversely, if λ is too high, γ will approach zero too rapidly, facilitating pruning but causing a significant reduction in model precision. Even with fine-tuning, the model might not achieve or maintain the original level of precision. A series of sparsity rates λ were established, and each sparsity rate underwent 100 rounds of pruning training. The statistical analysis of all results revealed that a pruning rate of 0.2 and a sparsity rate of 0.0018 yielded the best model performance.

**Fig 5:**
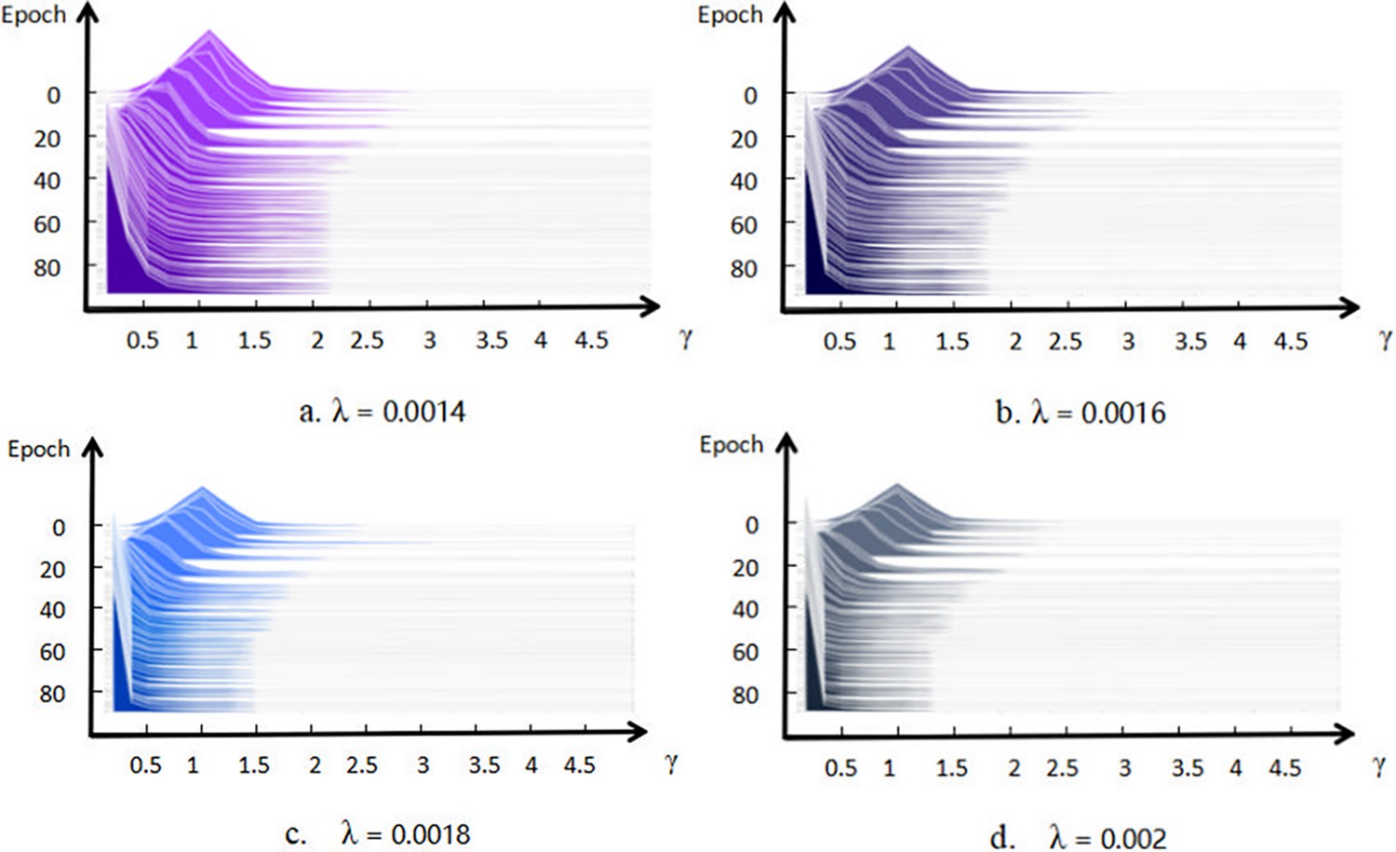
Distribution of scaling factor γ after 100 rounds of sparsity training with different sparsity rates λ.

Fig 5 illustrates part of the distribution of scaling factors γ under different sparsity rates λ. Prior to sparsity training, the scaling factor γ weights for each Batch Normalization (BN) layer are predominantly concentrated around 1, resembling a normal distribution. After sparsity training, an increasing number of BN layers exhibit scaling factor weights approaching zero. When the scaling factors of the sparse model approach zero, it indicates that the corresponding channels are relatively less important in the model. At a sparsity rate of 0.0014, the shaded area significantly decreases compared to the original model. At a sparsity rate of 0.0016, the scaling factor weights for each BN layer shift further to the left, with the shaded area diminishing further compared to the sparsity rate of 0.0014. At a sparsity rate of 0.0018, the plot shows a sharp decline in the slope of the scaling factor weights within a very narrow range. Increasing the sparsity rate to 0.0020 makes it challenging to distinguish changes in sparsity, but fortunately, performance data for models with different sparsity rates are still obtainable.

As shown in Table 2, before sparse training, the model’s mAP was 95.9%. With a sparsity rate of 0.0014, the mAP decreased by 1.5% compared to the original model. At a sparsity rate of 0.0016, the mAP decreased by 1%. At 0.0018, the mAP decreased by 0.2%, and at 0.0020, the mAP decreased by 0.7%. Therefore, this study adopted a sparsity rate of 0.0018.

**Table 2:** Model performance with different sparsity rates.

Sparsity	Pruning	mAP (%)	Parameters	Size (MB)	FLOPs (G)
0.0014	0.2	94.4	5405742	11.2	13.1
0.0016	0.2	94.9	5608658	11.6	13.8
0.0018	0.2	95.7	5893534	12.2	14.4
0.0020	0.2	95.2	6111366	12.6	14.8

### 3.4 Ablation experiment for the macro-detection algorithm model

To validate the effectiveness of the improved YOLOv5s-based detection algorithm for entire hydroponic Chinese flowering cabbage, an ablation experiment was conducted to analyze the impact of pruning and adding various modules on the model’s performance. As shown in Table 3, Scheme 1 involves pruning the YOLOv5s model. Scheme 2 builds upon Scheme 1 by introducing the GSConv module. Scheme 3 further extends Scheme 2 by incorporating the RFAConv module. Scheme 4 enhances Scheme 3 with the addition of the NAM module. Finally, Scheme 5 replaces the CIOU Loss with Focal-EIOU Loss, based on Scheme 4. The symbols "√" represent the adoption of the corresponding strategy in applying the module.

**Table 3:** Ablation experiment for the macro-detection algorithm model.

Scheme	Prune	GSConv	RFAConv	NAM	Focal-EIOU	mAP (%)	Size (MB)	Parameters		FLOPs(G)
YOLOv5s						95.9	14.4		7012822	15.8
1	√					95.7	12.2		5893534	14.4
2	√	√				95.7	11.5		5550434	14.0
3	√	√	√			96.0	11.9		5732834	14.5
4	√	√	√	√		96.3	11.9		5733426	14.5
5	√	√	√	√	√	96.7	11.9		5733426	14.5

As shown in Table 3, following the ablation experiment of the baseline network model, the model’s complexity is significantly decreased compared to the original network. In Scheme 1, parameters are decreased to 84.04% of the baseline model, with model size and FLOPs compressed from 14.4 MB and 15.8 G to 12.2 MB and 14.4 G, respectively. This reduction in complexity results in a mere 0.2% decrease in mAP. The decrease can be attributed to the reduction in redundant parameters, which leads to insufficient feature extraction for darker or occluded Chinese flowering cabbage, causing the mAP to drop. Scheme 2, which incorporates the GSConv module, achieves further reductions in parameters, model size, and FLOPs to 94.17%, 94.26%, and 97.22%, respectively, compared to Scheme 1. Despite a slight decrease, the mAP remains unchanged. The introduction of the RFAConv module in Scheme 3 results in a 0.3% increase in mAP compared to Scheme 2, compensating for the precision loss. This module addresses the issue of convolution kernel parameter sharing, effectively capturing critical feature information of Chinese flowering cabbage images and enhancing detection precision. Scheme 4, with a 0.3% increase in mAP over Scheme 3, further improves model precision. Although Schemes 3 and 4 exhibit slightly higher complexity compared to Scheme 2, Scheme 4 achieves a 0.6% improvement in precision. Scheme 5, by replacing CIOU Loss with Focal-EIOU Loss, boosts mAP to 96.7%. Overall, the improved model sees a 0.8% increase in mAP, an 18% decrease in parameters, a reduction in model size to 11.9 MB, and a reduction in FLOPs to 14.5 G.

**Fig 6:**
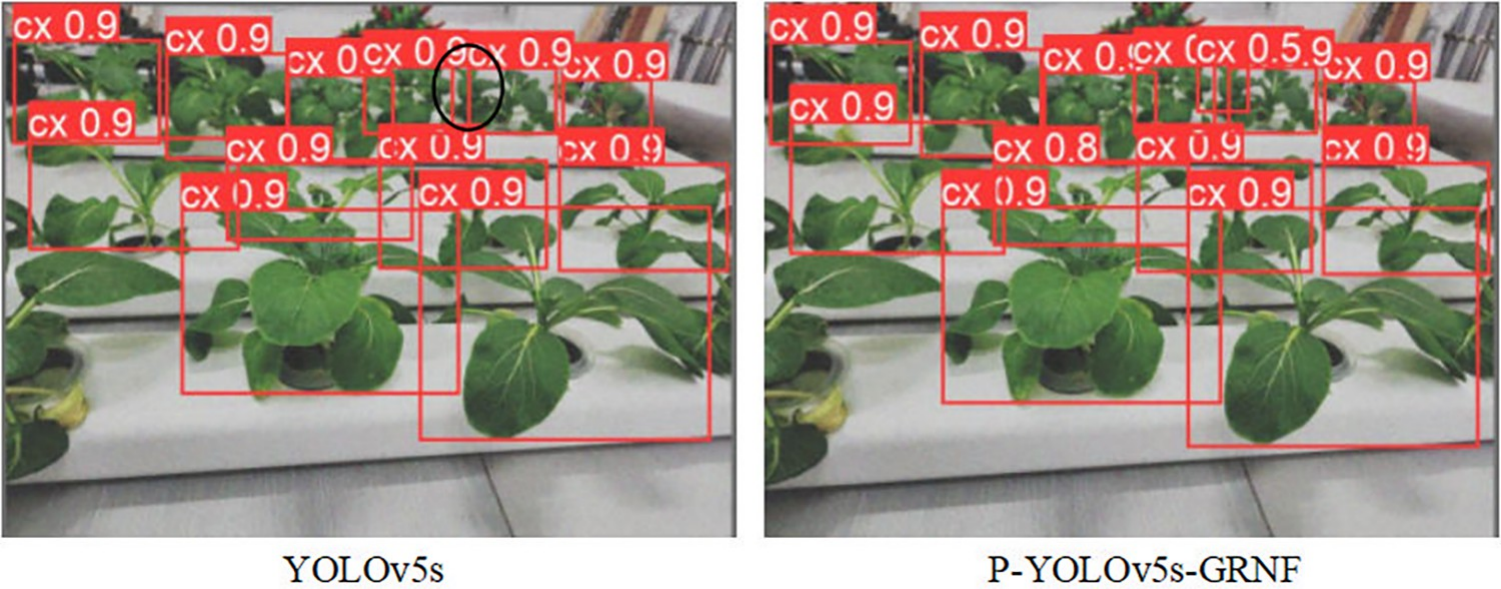
Comparison of detection results between YOLOv5s and P-YOLOv5s-GRNF.

To compare the detection effects of YOLOv5s and P-YOLOv5s-GRNF, parts of the visualization results are shown in Fig 6. The red rectangular box denotes the location of the Chinese flowering cabbage marked by the model, while the black oval circle denotes the location where the model has a false or missed inspection and is marked manually. Fig 6 shows the detection of Chinese flowering cabbage. In the first column of the hydroponic pipe, P-YOLOv5s-GRNF can completely identify all Chinese flowering cabbage but YOLOv5s misses one at the inner side of the right edge. Therefore, P-YOLOv5s-GRNF has a better actual detection effect.

### 3.5 Comparison experiment of the macro-detection algorithm model and different YOLOv5s lightweight models

To compare the detection performance of the P-YOLOv5s-GRNF model and other YOLOv5s lightweight models. Utilizing the same dataset of entire hydroponic Chinese flowering cabbage and maintaining consistent experimental conditions and parameters, the improved YOLOv5s model was evaluated against the YOLOv5s-Shufflenetv2, YOLOv5s-Mobilenetv3, and YOLOv5s-Ghost models.

As shown in Table 4, the YOLOv5s-Shufflenetv2, YOLOv5s-Mobilenetv3, and YOLOv5s-Ghost models have significantly decreased parameters, FLOPs, and model size, meanwhile, the FPS of YOLOv5s-Shufflenetv2 and YOLOv5s-Mobilenetv3 is higher than that of P-YOLOv5s-GRNF. However, their mAP decreases significantly, being 19.3%, 9.8%, and 3.1% lower than that of the model in this study, respectively. This results in a considerable number of missed detections, making them unsuitable for real-time detection of entire hydroponic Chinese flowering cabbage. In contrast, the model in this study, P-YOLOv5s-GRNF, achieves a balance between precision and speed, meeting the requirements for detecting entire hydroponic Chinese flowering cabbage.

**Table 4:** Comparison of different lightweight models.

Model	mAP (%)	Size (MB)	Parameters	FLOPs (G)	FPS
YOLOv5s-Shufflenetv2	77.4	2.0	842358	1.8	63.7
YOLOv5s-Mobilenetv3	86.9	3.1	1374732	2.3	56.2
YOLOv5s-Ghost	93.6	7.8	3679070	8.1	53.8
P-YOLOv5s-GRNF	96.7	11.9	5733426	14.5	55.6

### 3.6 Comparison experiment of the macro-detection algorithm model and different detection models

To assess the advantages of the improved P-YOLOv5s-GRNF model, this study compared it with existing mainstream models. Utilizing the same dataset of entire hydroponic Chinese flowering cabbage and maintaining consistent experimental conditions and parameters, the improved YOLOv5s model was evaluated against YOLOv5s, YOLOv6s [41], YOLOv7-tiny [42], and YOLOv8s [43]. The comparison results are shown in Table 5.

**Table 5:** Comparison result of different detection algorithms.

Model	mAP (%)	Size (MB)	Parameters (M)	FLOPs (G)	FPS
YOLOv5s	95.9	14.4	7.01	15.8	51.3
YOLOv6s	92.4	38.8	18.50	45.2	53.0
YOLOv7-tiny	93.5	12.3	6.01	13.0	52.9
YOLOv8s	96.0	22.5	11.13	28.4	56.5
P-YOLOv5s-GRNF	96.7	11.9	5.73	14.5	55.6

Table 5 reveals that the mAP of the P-YOLOv5s-GRNF model for detecting entire hydroponic Chinese flowering cabbage is increased by 0.8%, 4.3%, 3.2%, and 0.7% compared to YOLOv5s, YOLOv6s, YOLOv7-tiny, and YOLOv8s, respectively. Additionally, the model’s parameters have been decreased to 18% of those in YOLOv5s, 69% of those in YOLOv6s, 5% of those in YOLOv7-tiny, and 49% of those in YOLOv8s. The model size is decreased by 17.4%, 69.3%, 3.3%, 47.1% compared to YOLOv5s, YOLOv6s, YOLOv7-tiny, and YOLOv8s. Compared to YOLOv5s, YOLOv6s, and YOLOv7-tiny, the FPS increases by 4.3, 2.6, and 2.7, respectively, while it decreases by 0.9 compared to YOLOv8s. In summary, the improved P-YOLOv5s-GRNF model not only demonstrates superior detection performance but also proves to be more efficient for detecting entire hydroponic Chinese flowering cabbage.

### 3.7 Ablation experiment for the micro-localization algorithm model

To validate the effectiveness of the root area detection algorithm for hydroponic Chinese flowering cabbage based on the improved YOLOv5s model, an ablation experiment was conducted to analyze the impact of scaling adjust strategy and the addition of various modules on the model’s performance, as shown in Table 6. Scheme 1 involves adjusting the detection scale of the original model by adding a 160×160 detection layer and removing a 20×20 detection layer. Scheme 2 builds upon scheme 1 by introducing the BiFPN module. Scheme 3 further extends scheme 2 by incorporating the CA module. The symbols "√" represent the adoption of the corresponding strategy in applying the module.

**Table 6:** Ablation experiment for the micro-localization algorithm model.

Scheme	Scaling adjust	BiFPN	CA	mAP (%)	Size (MB)	Parameters	FLOPs (G)
YOLOv5s				88.6	14.4	7012822	15.8
1	√			90.6	5.8	2456726	15.3
2	√	√		91.7	5.8	2456736	15.3
3	√	√	√	92.6	5.8	2463416	15.3

As shown in Table 6, following the scaling adjustment strategy, the model’s complexity is significantly decreased compared to the original network. In scheme 1, parameters are decreased to 35% of the baseline model, a decrease of 65%. The model size is compressed from 14.4 MB to 5.8 MB, a reduction of 60%. FLOPs decrease from 15.8 G to 15.3 G. Moreover, this improvement results in a 2.0% increase in mAP. This can be attributed to adding the small object detection layer, which enables the model to detect even smaller objects, thereby enhancing precision. Furthermore, removing the large object detection layer reduces the model’s parameters without compromising precision. Scheme 2, which incorporates the BiFPN module, achieves a further increase in mAP of 1.1% compared to Scheme 1, with an almost negligible increase in parameters. The introduction of the CA mechanism module in scheme 3 results in the mAP increased to 92.6%. After improvement, the model’s mAP increased by 4.0%, while the parameters decreased by 65% and the model size decreased by 60%. FLOPs was decreased to 15.3G.

## 4 Detection and localization experiment

To assess the feasibility of the YOLOv5s-SBC model, it was integrated into the localization algorithm and combined with the Intel D435i depth camera to enable real-time detection and localization of the root area of hydroponic Chinese flowering cabbage. The camera captures real-time images of the hydroponic Chinese flowering cabbage, which are then preprocessed and input into the pre-trained YOLOv5s-SBC model for root area detection. The 2D coordinates of the bounding box’s center for the root area are subsequently converted into 3D coordinates within the camera’s coordinate system. The system provides real-time information on the detected target’s position, including the bounding box, label, and confidence score.

**Fig 7:**
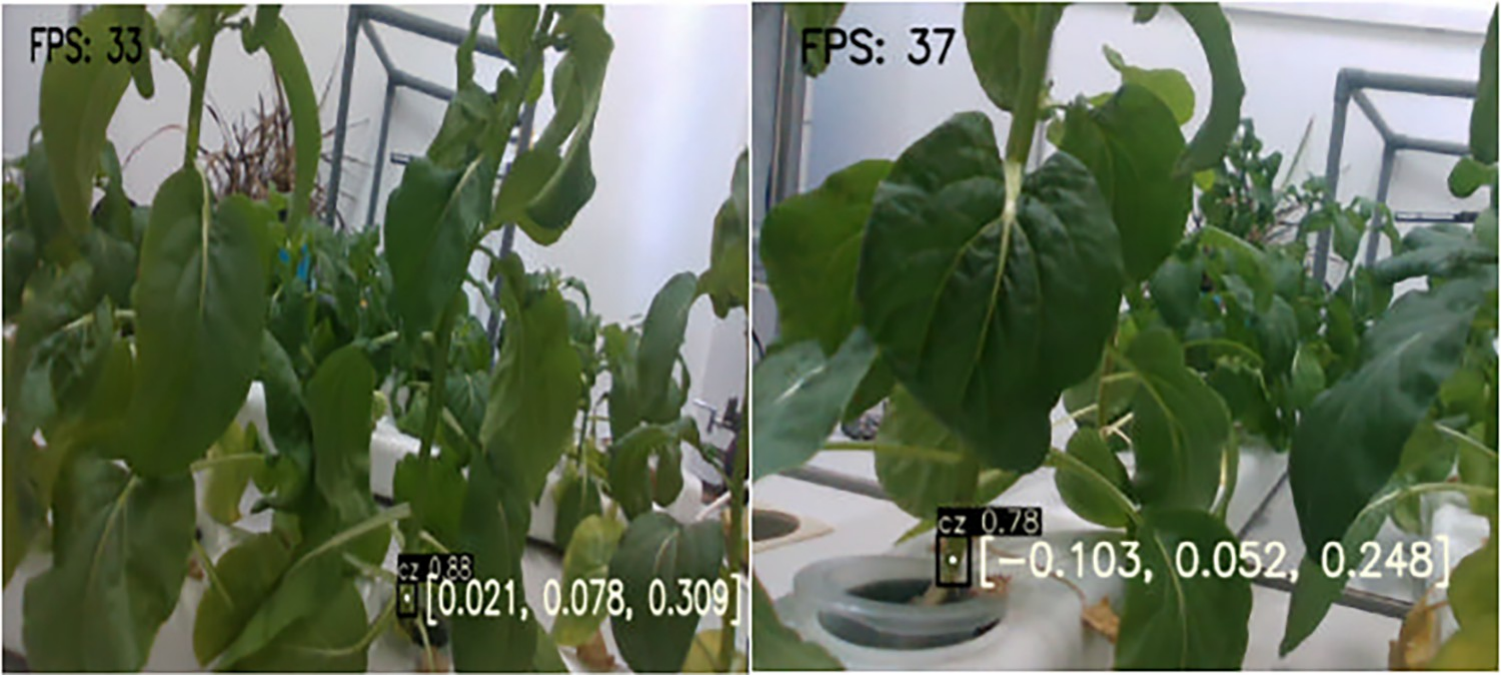
Single Chinese flowering cabbage detection and localization.

As shown in Fig 7, the detection and localization results for a single Chinese flowering cabbage using the YOLOv5s-SBC model are presented. The black boxes in the figure represent the automatically generated detection boxes, with confidence scores of 0.88 and 0.78. The coordinates [0.021, 0.078, 0.309] and [-0.103, 0.052, 0.248] indicate the 3D coordinates within the camera’s coordinate system. During the experiment, the success rate for detection and localization of a single Chinese flowering cabbage is 100%.

**Fig 8:**
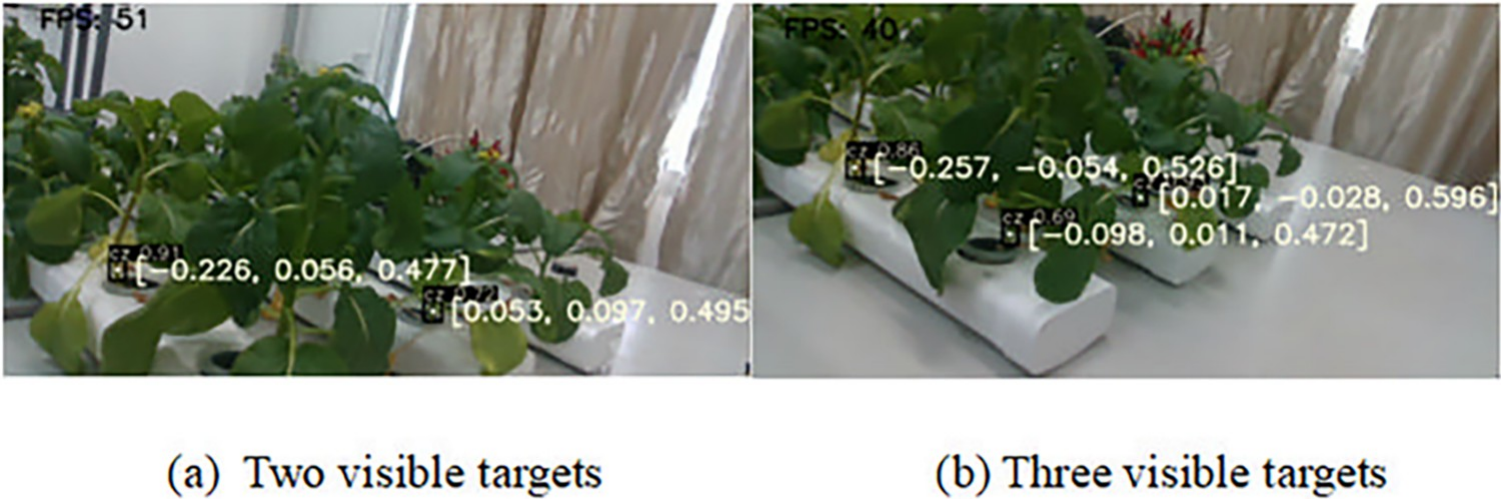
Multiple Chinese flowering cabbage without leaf occlusion detection and localization.

As illustrated in Fig 8, the detection and localization results of multiple Chinese flowering cabbages without leaf occlusion using the YOLOv5s-SBC model are presented. In Fig 8A, the automatically generated black boxes indicate the detected targets, with two visible root area of Chinese flowering cabbage successfully identified, resulting in a detection success rate of 100%. Fig 8B features three targets, all of which were detected, also achieving a 100% success rate. Average detection success rate of repeatedly experiments for multiple Chinese flowering cabbages without leaf occlusion is 100%.

**Fig 9:**
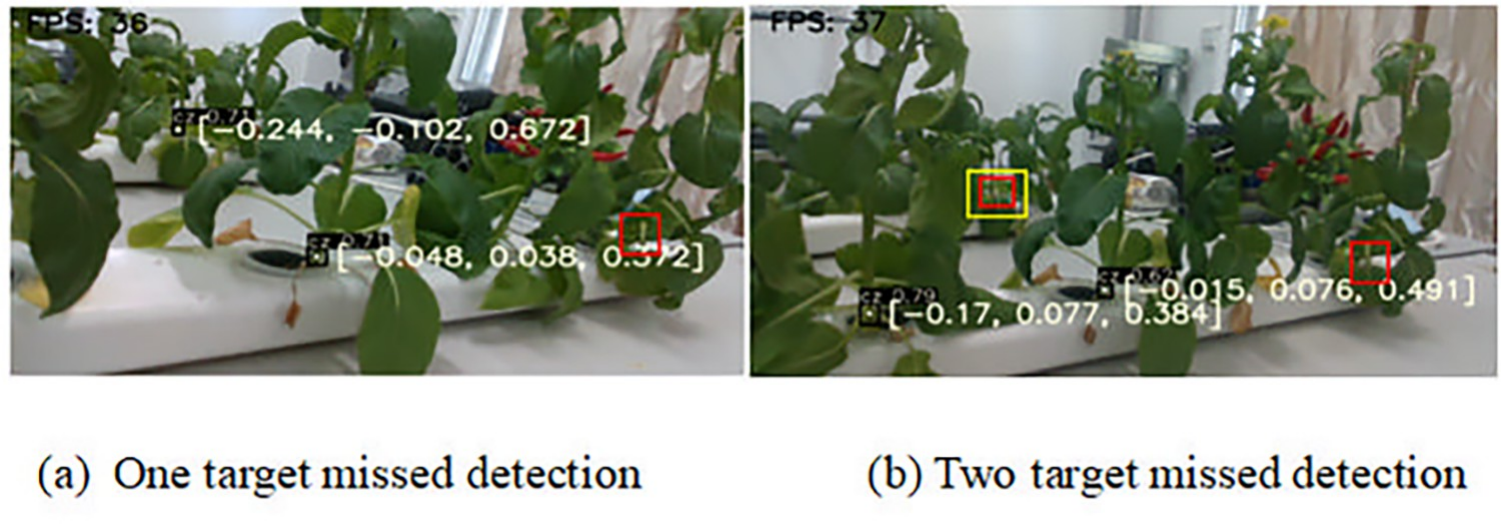
Multiple Chinese flowering cabbage with leaf occlusion detection and localization.

Fig 9 presents the detection and localization results of multiple Chinese flowering cabbages with leaf occlusion using the YOLOv5s-SBC model. In Fig 9A, red boxes highlight missed detections, with three targets in total—two detected and one missed. Fig 9B shows yellow boxes indicating missed detections due to distance from the camera; out of four targets, two were detected and two missed. Average detection success rate of repeatedly experiments for this scenario is 78.4%. The low success rate is attributed to leaf occlusion and distance, which hinder clear imaging. To improve detection, closer proximity and camera angle adjustments on the robotic arm are essential during harvesting, allowing for unobstructed views of the root area for accurate target recognition.

## 5 Conclusion

To address the challenges of detection and localization for hydroponic Chinese flowering cabbage, this study proposes a two stages algorithm comprising macro-detection and micro-localization. The macro-detection algorithm, termed P-YOLOv5s-GRNF, focuses on detecting the entire hydroponic Chinese flowering cabbage. It incorporates strategies such as pruning techniques, the GSConv, RFAConv, NAM, and the Focal-EIOU Loss module to enhance recognition efficiency and precision. The micro-localization algorithm, named YOLOv5s-SBC, targets the detection of the root area of Hydroponic Chinese flowering cabbage. It employs techniques including the addition of a 160×160 detection layer, removal of a 20×20 detection layer, introduction of a weighted BiFPN structure, and application of the CA mechanism to improve localization efficiency and precision. The following conclusions were obtained through the experiments described in this study:

(1) Compared to YOLOv5s, the improved P-YOLOv5s-GRNF model shows a 0.8% increase in mAP, an 18% reduction in parameters, a decrease in model size to 11.9MB, and a reduction in FLOPs to 14.5G, meeting real-time detection requirements for the entire hydroponic Chinese flowering cabbage. Meanwhile, the YOLOv5s-SBC model achieves a 4.0% increase in average precision, a 65% reduction in parameters, a 60% decrease in model size, and a reduction in FLOPs to 15.3G.

(2) The P-YOLOv5s-GRNF model outperforms YOLOv5s, YOLOv6s, YOLOv7-tiny, and YOLOv8s on the complete hydroponic Chinese flowering cabbage dataset, with average precision improvements of 0.8%, 4.3%, 3.2%, and 0.7%, respectively. Additionally, compared to other lightweight YOLOv5s models, the P-YOLOv5s-GRNF model achieves mAP improvements ranging from 3.1% to 19.3%.

(3) Combined with a depth camera, the YOLOv5s-SBC model achieved a 100% detection success rate for single Chinese flowering cabbages and multiple cabbages without leaf occlusion. However, the success rate is significantly lower for multiple cabbages with leaf occlusion. Therefore, adjusting the harvesting angle and distance with the robotic arm is essential for improved detection.

The improved model demonstrated good performance in the detection and localization experiments, providing theoretical support for the automation of hydroponic Chinese flowering cabbage harvesting.
